# Investigating patient-specific mechanisms of change in SET vs. EFT for depression: study protocol for a mechanistic randomized controlled trial

**DOI:** 10.1186/s12888-021-03279-y

**Published:** 2021-06-02

**Authors:** Sigal Zilcha-Mano, Ben Shahar, Hadar Fisher, Tohar Dolev-Amit, Leslie S. Greenberg, Jacques P. Barber

**Affiliations:** 1 The Department of Psychology, University of Haifa, 31905 Haifa, Mount Carmel Israel; 2 The Paul Baerwald School of Social Work and Social Welfare, Hebrew University, Jerusalem, Israel; 3 Department of Psychology, York University, Toronto, Canada; 4 The Gordon F. Derner School of Psychology, Adelphi University, Adelphi, USA

**Keywords:** Personalized treatment, Supportive-expressive treatment, Emotion-focused treatment, Mechanisms of change, Insight, Emotional processing

## Abstract

**Background:**

Major depressive disorder (MDD) is the leading cause of disability worldwide and one of the most heterogeneous mental health disorders. Although there are effective treatments for MDD, about 50% of patients do not respond to treatment. One of the greatest challenges in improving current treatments is identifying the mechanisms responsible for therapeutic change in MDD. The proposed study aims to identify patient-specific mechanisms of change in two treatments for MDD by investigating whether subpopulations of patients differ in the mechanisms of change that operate when receiving a given treatment. Based on theories of targeting weakness and building on strength, we will examine whether the mechanism of change operating when a treatment is provided depends on whether the treatment targets the patient’s strength or weakness.

**Method:**

To test our hypothesis that two treatments, supportive-expressive treatment (SET) and emotion-focused treatment (EFT), differ in their mechanisms of change and to explore whether focusing on the patient’s strength or weakness will result in better treatment outcome, we conduct a mechanistic randomized controlled trial. One hundred and twenty-four individuals diagnosed with MDD are randomized to 16 sessions of either SET or EFT. The two treatments are theorized to differ in their main mechanism of change: SET places emphasis on insight as its main mechanism of change, and EFT places emphasis on emotional processing. Both can serve as strength- or weakness-focused treatments, based on the patient’s baseline levels of insight and emotional processing. The primary outcome is the Hamilton Rating Scale for Depression. Additional measures include self-report measures and clinical interviews, hormonal, motion, acoustic, physiological, and neuroimaging assessments, performance on cognitive tasks, and narrative material (collected from the sessions and interviews).

**Discussion:**

The RCT will expand our understanding of mechanisms of change in psychotherapy, from one-size-fits-all to patient-specific mechanisms of change. By informing therapists about which of the two approaches is most effective with patients based on their baseline characteristics, the RCT will contribute to progress toward personalized treatment.

**Trial registration:**

clinicaltrials.gov Identifier: NCT04576182 submitted on October 1st 2020. Funding: The Israel Science Foundation. Trial status: Recruitment is ongoing.

## Background

Major depressive disorder (MDD) is the leading cause of disability worldwide, and a main contributor to the overall global burden of disease [1]. Many active treatments for MDD are available, differing in their underlying mechanisms theorized to drive therapeutic change, including a variety of cognitive behavioral, psychodynamic, and emotion-focused therapies [2, 3]. Yet, they do not seem to differ in their efficacy: they all show a treatment response rate at about 50% for the “average patient” [4]. Despite the absence of difference in efficacy between distinct treatments for the average patient, large variability is present within treatments, with some subpopulation of patients showing great ability to benefit from a given treatment, whereas others are less able to do so, and may even deteriorate [5, 6]. Better understanding of the mechanisms that bring about therapeutic change can help us devise and deliver better treatments by intensifying and refining active therapeutic components [7].

For decades, theoreticians and researchers sought to understand the mechanisms that bring about therapeutic change, garnering important knowledge [8, 9]. The vast majority of studies used the classical design for investigating mechanisms of change: randomized controlled trials (RCTs) that randomize patients to one of two or more treatments, and test whether each treatment affects outcome for the “average patient” through its theorized treatment-specific mechanism of change. Using such designs, several central mechanisms were the focus of investigation, including *insight* [2, 10] and *emotional experiencing, processing,* and *regulation* [11, 12]. A range of studies found that improvements in insight, that is, patients’ gains in understanding of their maladaptive patterns, especially maladaptive relationship patterns, has a significant effect on treatment outcome [13–15]. Similar findings were reported for improvements in emotional processing, generally defined as improved awareness of emotional experiences, and of the ability to label, regulate, and transform emotions [16, 17]. Improving the ability to experience adaptive emotions and acquiring better emotion regulation capacities have been related to better treatment outcome (e.g., [18, 19]). Meta-analyses and systematic reviews suggest, however, that findings on the effects of improvement in mechanisms on outcome are less consistent than one may have anticipated based on the theoretical models of change of each treatment [8]. Lorenzo-Luaces et al. [20] articulated this problem well in their article entitled: “It’s complicated.”

We suggest that the mixed findings are, at least in part, the result of following the classical model of investigating mechanisms of change, and the corresponding study design. This design assumes that all patients randomized to a given treatment condition have the same potential to benefit from it, so that each individual that received an adequate amount of treatment will show improvement in the treatment-specific mechanism (e.g., will gain insight, will manage emotions better). Yet, if different subpopulations of patients have different potential to show improvement in a given mechanism, even if receiving an adequate amount of treatment, this assumption is no longer true. Emerging empirical and simulation work suggests that subpopulations of patients differ in their potential to show improvement in any given mechanism [5] and to benefit from treatments that are based on distinct mechanisms of change [21]. This expanding literature is consistent with recent efforts toward personalized treatment, which is based on the core principle that although many treatments are effective at the population level, each subpopulation may benefit most from a given treatment or “family” of treatments (e.g., treatments based on one’s weakness [22]). Building on theorized differences between the active mechanisms of treatments (e.g., some treatments focus on improving emotional processing, others on gaining insight), and on personalized treatment approaches arguing that individuals differ in their potential to benefit from any given mechanism (e.g., some individuals benefit most from greater ability to process emotions, others from gaining insight), we argue that better fit between patients and their assigned treatments, based on their mechanisms of change, will result in optimizing treatment outcome [23, 24].

To identify the mechanisms of change most critical for each subpopulation of patients, we propose that the two most researched questions in psychotherapy, *why* treatment works and *for whom,* should be integrated [23]. Although the questions *for whom* a given treatment works (identifying moderators), and *why* (identifying mediators) have evolved over time mostly as separate streams, it is important to integrate them. They are theoretically closely related, because moderators suggest that distinct processes may be involved for different subpopulations that benefit differently from the intervention (e.g., moderated mediation models [7, 25];). Integrating these two questions can lead to high-resolution psychotherapy research that better captures the complexity and richness of the process of therapeutic change. Thus, the question becomes: What makes a given treatment effective for a subpopulation of patients? The focus shifts to patient-specific mechanisms of change.

To focus on patient-specific mechanisms of change, one should first identify the subpopulations expected to benefit most from each given mechanism, and then test the mechanism for the specific subpopulation. Regarding the first step, the literature distinguishes between two approaches [26]: data-driven and theory-driven. In the data-driven approach, machine learning methods have been effectively used in recent years to identify moderators [21]. These methods require very large datasets to adequately implement systematic data-driven exploration, and, at present, they are conducted mainly as secondary post hoc analyses. In the theory-driven approach, meta-theories suggest which subpopulation will benefit most from each treatment or groups of treatments. These meta-theories complement the treatment-specific theories, and provide an organizing principle by which patients can be matched to treatment. Typical examples are the meta-theories building on patients’ weaknesses or capitalizing on their strengths. Based on strengths and weaknesses in the areas targeted by different treatments, these theories argue that patients are not uniformly receptive to the mechanisms underlying distinct treatments, and may respond to these strategies differently [22, 27, 28]. Pre-treatment strengths and weaknesses in the targeted mechanisms of change can be used either by choosing to compensate for relative weaknesses or to build on relative strengths by capitalizing on them. The theory of weakness proposes a compensation model whereby treatment will result in better outcomes if it targets the relative deficits of the patients, helping them acquire skills and capacities that they do not yet possess. The theory of strength proposes targeting the strengths of the patients, co-opting the patient’s most adaptive capabilities of perceiving and acting in the world [27].

Although these meta-theories received much attention and raised great interest in clinical practice, only a handful of studies have investigated empirically their utility in improving outcome (e.g., [22, 27]), and no study, to our knowledge, explored the way they operate to affect outcome. Studies examining the utility of these meta-theories in improving outcome produced mixed results, some showing support for targeting weakness [22], the others for capitalizing on strength (e.g., [27, 29]). We propose that both theories may be critical in understanding patient-treatment fit, but they operate through distinct mechanisms. The theory targeting weakness may operate through theory-specific mechanisms of change. For example, when patients who are low on insight receive a treatment based on insight as its core mechanism of change [30], the patients may show gains in insight, which in turn brings about reduction in symptoms [31]. In the same vein, when patients who are low on emotional processing (they have a low ability to be aware, express, and make meaning of their emotions) receive treatment that focuses on emotional processes as its core mechanism of change [32], they may show gains in emotional processing, which in turn can bring about reduction in symptoms [33].

What happens when a patient receives a treatment that is based on the patient’s strength as its main mechanism of change? In the example of insight, the patients receive a treatment based on insight, but they may show no or very little change in insight because of an already high level of performance in this area (ceiling effect). Yet, it may still be effective to work on the patient’s strengths, not because of the theory-specific mechanism of change (boosting insight and emotional processing), but because alternative mechanisms may play a role. Promising candidates for such alternative mechanisms are the common mechanisms, which have been shown to contribute to outcome across treatments [34]. The most researched common mechanism is the working alliance between patients and their therapists, generally defined as the emotional bond established in the therapeutic dyad and the agreement between patient and therapists concerning therapy goals and the tasks necessary to achieve them [35–37]. When working on strengths, the therapists may adopt techniques that are less intended to confront the patients with a different way of perceiving and reacting to the world, and more naturally support the patient’s own adaptive patterns of experiencing and reacting. Such supporting work in treatment may help therapists create a strong emotional bond with their patients, who may more readily perceive the therapists as acting for their benefit. Patients may also find it easier to agree with the therapists on the tasks and goals of treatment, which mirror their own assets. Thus, capitalizing on patients’ strengths may bring about therapeutic change through the mechanism of strengthening the alliance.

**Fig. 1:**
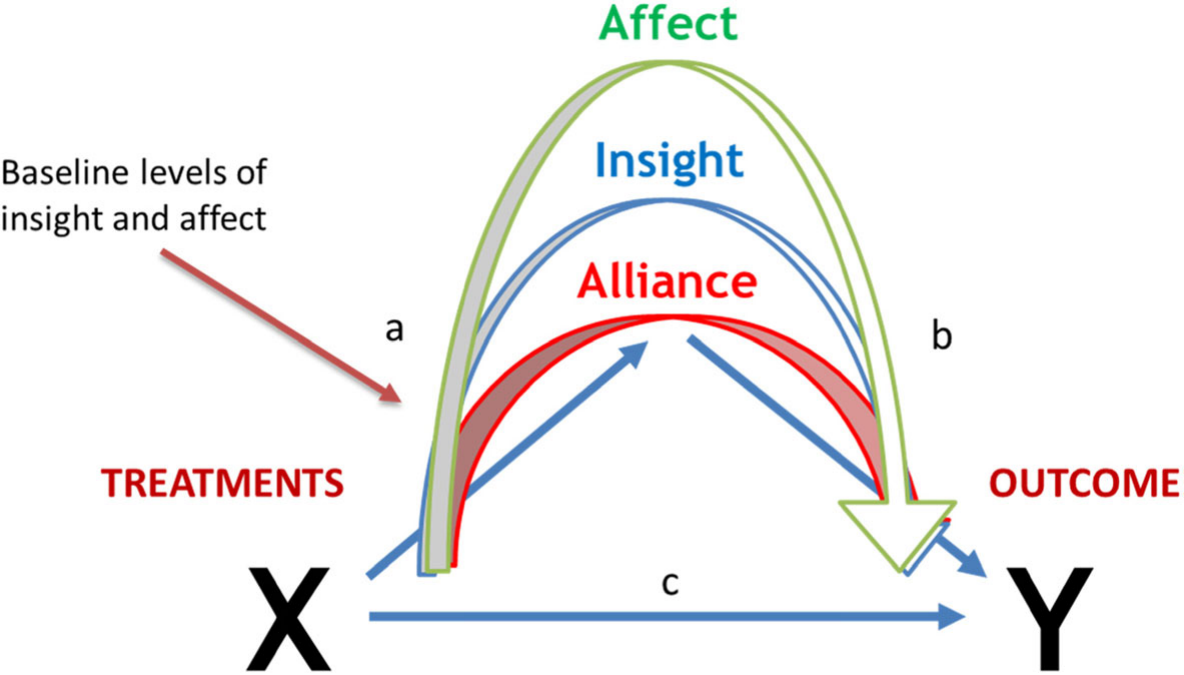
The proposed moderated-mediation model. The figure presents the three potential mediators: insight, emotional processing, and alliance, as well as the moderators: baseline insight and emotional processing

The primary objective of the proposed study is to explore patient-specific mechanisms of change, by investigating whether subpopulations of patients differ in their mechanism of change when receiving a given treatment. Based on the theories of targeting weakness and building on strength, we argue that the mechanism that operates when a treatment is administered depends on the potential of the subpopulation to benefit from the treatment-specific mechanism of change, according to the subpopulations’ strengths or weaknesses. The proposed moderated-mediation model is presented in Fig. 1. *The first goal* is to investigate the mechanisms of change that are activated when individuals receive a treatment that targets their weakness; *the second goal* is to investigate the mechanisms activated when the treatment capitalizes on their strength.

### Study hypotheses

1. For patients receiving treatment focusing on their weakness rather than their strength, the treatment operates in a manner consistent with the classical mediation model: a therapy focusing on a specific mechanism is expected to produce a change in it, which in turn results in improvement in outcome.
  - Patients with low levels of insight receiving treatment designed to improve insight rather than emotional processing will show a rise in insight, which will result in improvement in outcome.
  - Patients with low levels of emotional processing capacity receiving treatment designed to improve emotional processing rather than insight will show improvement in emotional processing, which will result in improvement in outcome.
2. For patients receiving treatment focusing on their strength rather than weakness, the treatment operates according to an alternative mediation model: the treatment is expected to strengthen the alliance, which in turn results in improvement in outcome.
  (c) Patients with high levels of insight receiving treatment designed to improve insight rather than emotional processing will show strengthening in alliance, which will result in improvement in outcome.
  (d) Patients with high levels of emotional processing capacity receiving treatment designed to improve emotional processing rather than insight will show strengthening in alliance, which will result in improvement in outcome.

## Method

### Study design

The proposed trial aims to investigate the different mechanisms underlying SET vs. EFT, and follows the requirement for an RCT with a design of two parallel groups. Each condition can be considered as a control group for the other [38]. Participants will be allocated into one of two groups, SET and EFT, with an assignment ratio of 1:1. To ensure balance across age, sex, family status, baseline 17-item Hamilton Depression Rating Scale (HRSD), and personality disorders, a minimization algorithm will be used. Only the therapists and their supervisors will be exposed to participant allocation, while the rest of the research team will be blind to the patients’ assignment to treatment. The study protocol follows the Standard Protocol Items: Recommendations for Interventional Trials (SPIRIT) checklist [39].

### Participants

**Fig. 2:**
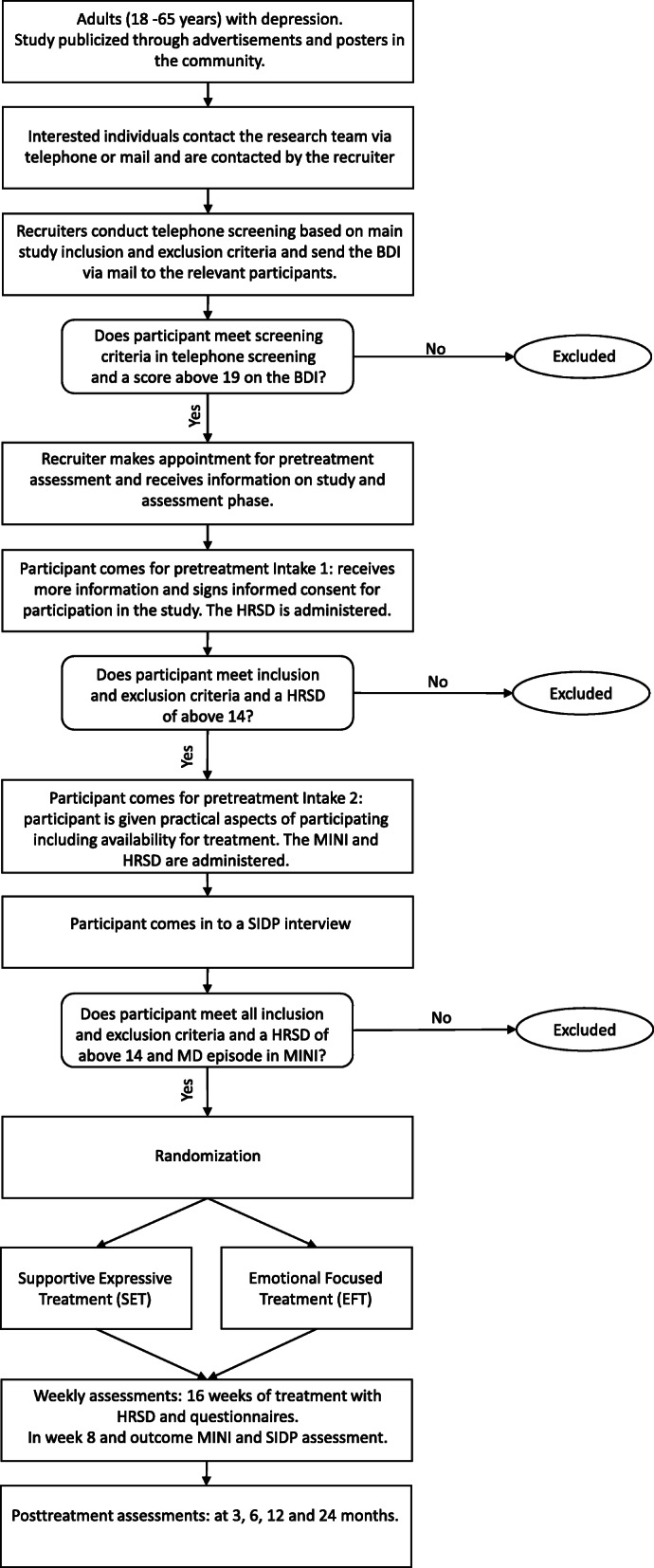
Flow of participants in the study

Participants will be one hundred and twenty-four patients diagnosed with MDD who live in Israel. Recruitment will take place through advertisements, offering free treatment at the University of Haifa Psychotherapy Research Lab clinic (Fig. 2). The sample size was chosen to provide adequate power to test our hypotheses.

**Inclusion criteria:** (a) current MDD based on the Mini International Neuropsychiatric Interview, MINI 7.0.2 (Structured Clinical Interview for DSM5 [40, 41]), and scores above 14 on the 17-item HRSD [42] at two consecutive assessments, one week apart; (b) for participants using psychiatric medication, the dosage must be stable for at least three months before the beginning of the study, and they will be asked to maintain stable dosage during the treatment; (c) age between 18 and 65 years [43]; (d) Hebrew language proficiency; (e) provide written informed consent.

**Exclusion criteria:** (a) current or past schizophrenia or psychosis, bipolar disorder, or severe eating disorder, demanding close medical monitoring; (b) history of organic mental disease; (c) current risk of suicide or self-harm (HRSD suicide item > 2); (d) current substance abuse disorder; (e) being currently in psychotherapy.

### Treatments

Patients will be randomized to 16 50-min sessions of either SET or EFT. SET is a time-limited psychodynamic therapy adapted for depression. It includes the use of supportive techniques, such as affirmation and empathic validation, as well as more expressive techniques, such as interpretation, confrontation, and clarification (SE [30, 44]). EFT is a time-limited therapy combining client-centered elements (unconditional positive regard, congruence, and empathy) with marker-guided experiential interventions designed to facilitate and deepen emotional processing [32, 45]. For both treatments, we will use comprehensive treatment protocols. For SE treatment, we will use the Luborsky [30, 44] manualized treatment; for EFT, the Greenberg and Watson’s manualized treatment [45].

### Therapists

Both SET and EFT will be carried out by therapists experienced in that treatment. Before starting to treat patients, therapists will receive an intensive training on the relevant treatment, and will treat a pilot case coded for adherence to ensure adequate levels of proficiency. Therapists will participate in weekly group supervisions during the whole trial. The supervision will make extensive use of videotaped sessions for feedback. The primary developer of EFT and an international expert in SE, with more than 25 years of experience in this treatment, will be involved in and will accompany the process of supervision.

### Fidelity check

To ensure that therapists adhere to the manual and that they are competent in the technique, they will attend weekly supervisions in which videotaped sessions will be viewed to identify and analyze instances of deviation from the manual. In addition, each therapy session will be videotaped, and the therapists rated for adherence and competence by external evaluators. In the SET condition, adherence and competence will be evaluated using the Penn Adherence-Competence Scale (PACS [46]). The PACS consists of three subscales: supportive component, expressive component, and general therapeutic behaviors. The raters will be supervised by an international expert on the use of the PACS, with vast experience in using the PACS in RCTs on SE treatment. In cases of low adherence or insufficient competence, the therapist and the supervisor will be informed and relevant supervision tools will be deployed (e.g., [47, 48]).

In the EFT condition, adherence and competence will be evaluated using the Person-Centered and Experiential Psychotherapy Scale (PCEPS [49]). The PCEPS assesses adherence in empathic attunement and in several EFT task interventions (two-chair intervention for self-criticism and empty-chair dialogue for unresolved feelings). The research team will receive ongoing weekly supervision from Leslie Greenberg, the primary developer of EFT. In cases of low adherence or insufficient competence, relevant supervision tools will be deployed.

### Procedure and randomization

Recruitment will be through self-referral, based on advertisements in local media, and online publications (social media, etc.). Interested individuals will be instructed to apply by email or by phone. In an initial phone screening, members of the research team will inform the applicants about the study details and procedure, and about the inclusion and exclusion criteria, and evaluate indications for depressive symptoms. To evaluate severity of depressive symptoms, applicants who meet the eligibility criteria and consent to participate in the research intake procedure will be asked to complete the Beck Depression Inventory (BDI-II [50]). A score of at least 19 points on the BDI-II was determined as the cut-off for proceeding to the next phase of the study. Applicants who score 19 points and above on the BDI-II will be contacted to schedule an initial intake meeting, during which the researchers will provide detailed written and oral information about the procedure of the planned study and encourage applicants to ask questions. The researchers will inform applicants that all treatment sessions will be videotaped and that they have the right to withdraw from the research at any time. The researchers will also inform applicants about the full research procedure and assessments, its safety, and possible risks. A first face-to-face assessment meeting will be scheduled only for applicants who agree to participate and sign the informed consent form. At the first assessment meeting, participants will be asked to complete a battery of questionnaires that includes demographic information, concurrent medication, comorbid conditions, several personality and interpersonal characteristics, and baseline symptom self-reports. To measure the presence and severity of baseline MDD symptoms, an HRSD interview will be conducted by trained interviewers. At the second assessment meeting, the HRSD will be administered again. In addition, the MINI will be administered to measure the presence and severity of baseline MDD symptoms and comorbid conditions. At the second assessment meeting, participants will also provide saliva samples. Following the second interview, a third face-to-face meeting will be scheduled, in which a different interviewer will assess comorbid personality disorders using the Structured Clinical Interview for DSM- IV Personality Disorders (SIDP [51]).

The HRSD, the MINI, and SIDP interviews will be conducted by independent, rigorously trained research interviewers. After completing 2 m of extensive training and successfully achieving high reliability, the interviewers will observe another interviewer at work. Only then will they begin to administer the measures, first with a trained interviewer observing them in the first several weeks, and later by themselves. During the trial period, the reliability of the interviewers will be evaluated weekly.

Patients who meet the eligibility criteria and agree to participate in the study will be randomly assigned to either the SET or the EFT therapy condition. Randomization will be performed by an independent institution, not involved in the study. Applicants who have not met the inclusion criteria or have met the exclusion criteria and therefore are not eligible for the study, but request psychotherapy, will be referred to alternative care. Research team members, including the researchers, assessors, and interviewers will be blinded to treatment assignment. By necessity, only the therapists and clinical supervisors will be exposed to treatment allocation. To minimize bias that could be derived from incidences in which blindness is broken, the following strategies will be used: (a) patients, as well as all process and outcome evaluators will be blind to treatment assignment; (b) therapists will be frequently reminded to make sure that they conceal treatment assignment from any member of the team; (c) patients and outcome evaluators will be asked to speculate to which treatment the patient was assigned, making it possible to examine bias in the analyses; and (d) all outcome evaluator interviews will be videotaped, and 30% of the tapes will be randomly assigned and rerated by independent raters. In case blindness is broken, all succeeding assessments will be conducted by an alternative evaluator.

During the active phase of treatment, the HRSD and self-report questionnaires will be administered weekly. Participating patients will be asked to complete self-report measures assessing their symptoms (e.g., BDI-II) before each session, and self-report measures assessing treatment processes following each session. Level of insight and emotional processing will be measured at six time points during therapy. Before the beginning of therapy and every fourth session during the course of treatment, patients will be asked to complete cognitive tasks and to provide saliva samples. In addition to baseline assessment, the MINI and SIDP will be measured at weeks 8 and 16. Patients who choose to withdraw from treatment before the end of the active treatment phase will receive a dropout questionnaire. Their therapists will also be asked to complete a dropout questionnaire [52].

After completion of the active phase of the treatment (16 weeks), follow-up assessments will be scheduled at 3, 6, 12, and 24 months after the end of the active phase. The follow-up assessment sessions will include HRSD, MINI, and a battery of self-report questionnaires. At 6 and 12 months, the SIDP will also be administered. To avoid bias, patients will be asked not to join other psychotherapeutic programs and not to change medication dosages.

### Measures

Severity of depressive symptoms, measured using the HRSD [42], will be the primary outcome measure of the trial. The HRSD is a 17-item measure administered by clinical interviewers. Depressive symptoms severity is calculated by summing up all 17 items. Secondary outcomes will be assessed using, among others, self-reported symptoms (i.e., BDI [50]; Beck Anxiety Inventory, BAI [53]; Outcome Questionnaire-30, OQ-30 [54]), interpersonal functioning (i.e., Interpersonal Problems Inventory, IIP-32 [55]), and quality of life (Quality of Life Enjoyment and Satisfaction, Q-LES-Q [56]). Process measures will be assessed using the Self-Understanding of Interpersonal Patterns Scale, (SUIP [13]), the Emotion Approach Coping Scale (EACS [57]), the Emotion Regulation Questionnaire (ERQ [58]), the Difficulties in Emotion Regulation Scale (DERS [59]). We will also assess the working alliance (Working Alliance Inventory, WAI [60]), affect (Positive and Negative Affect Scale, PANAS [61]), and therapeutic interventions (Multi-theoretical List of Therapeutic Interventions, MULTI [62]). We will use validated versions of all questionnaires in Hebrew.

### Ethical considerations

The study design, procedure, and informed consent form were approved by the ethics committee of the University of Haifa (approval number: 395/19, Date: 10/30/2019). Any modifications of the protocol will be reported to the committee. As detailed above, participants will be given comprehensive information about the study procedure both orally and in writing. The information describes the possible implications of their participation, including potential risks, inconvenience, and benefits. In the event of a significant adverse incident (e.g., severe suicide risk), the RISK protocol will be activated. Specifically, a special committee including three licensed clinical psychologists trained to deal with such situations will immediately take charge.

### Data management

To warrant confidentiality of the data at all stages of the research process, an elaborated data management plan is deployed. A data monitoring committee will supervise data collection. An identification number will be assigned to each participant on enrollment and will be used for data registration. The correspondence list will be kept securely in a password-protected computer. After completion of the trial, the raw data will be kept in a locked drawer in a locked room at the University of Haifa Psychotherapy Lab. After the dataset is de-identified and anonymized, researchers who will be approved by the steering committee will be able to access the dataset.

### Data analysis and power analysis

Analyses will be conducted on the intention-to-treat sample, using moderated-mediation models. The main analyses will focus on the main outcome measure (HRSD) and on the measures for each of the three mechanisms. Characteristics of the treatment groups will be described at baseline. Insight and emotional processing at baseline will serve as the moderators. We will examine the proposed moderated-mediation model for each mechanism separately, in a series of multilevel models [25]. According to the literature, in the type of analyses we conduct, it is possible to use the level of a measure at baseline as a moderator, and changes in the same measure during treatment as a mediator [26]. Therapist effects will be estimated as random effects in all models.

The sample size was chosen to provide adequate power to test our hypotheses. To estimate the final sample size for the study, we first estimated the required sample size for the first step, predicting the mechanism using Monte Carlo simulations in the R package “powerlmm” [63]. Assuming alpha = 0.05 and a medium effect size (Cohen’s D = 0.6), the simulations indicated a need for a sample size of 120 participants to ensure a power of at least 0.80. Next, using the same approach, we estimated the required sample size for the second step of the proposed moderated-mediation model. Assuming alpha = 0.05 and a medium effect size (Cohen’s D = 0.6) for the association between a mechanism and treatment outcome, the simulations indicated a need for a sample size of 116 participants to ensure a power of at least 0.80. Finally, we calculated the required sample size for the entire moderated-mediation model, using Monte-Carlo simulations and previously assumed effect sizes for each step, resulting in 124 patients.

### Dissemination policy

Study results will be disseminated to the public at academic conferences and in peer-reviewed journals. The steering committee will determine authorship of the planned primary and secondary publications. The order of the authors will be decided based on the contributions of each member.

## Discussion

MDD is the leading cause of disability worldwide [1]. Several treatments for MDD have been found to be similarly effective and more effective than control conditions, including supportive-expressive treatment [64] and emotion-focused treatment [65]). Yet, about half the patients receiving any treatment for MDD fail to show adequate response. Although there is low variance between distinct psychotherapies for MDD, there is high variance within each psychotherapy condition. Secondary post hoc analyses on RCT datasets suggest that different subgroups of MDD patients respond best to different treatments, highlighting the need for progress toward personalized treatment for MDD [21, 22]. Currently, there are few evidence-based tools to guide clinicians in patient assignment to their optimal treatment.

The present research makes several important contributions: (a) Theoretical contribution to *psychotherapy* research. This is the first study to explore patient-specific mechanisms of change by integrating the two most researched questions in psychotherapy research: why treatment works and for whom. This conceptual convergence can lead to higher-resolution psychotherapy research that better captures the complexity and richness of the process of therapeutic change. (b) Theoretical contribution to *psychopathology* research. Identifying unique mechanisms of change for given subpopulations of patients can advance our understanding of subtypes within MDD diagnosis and isolate the differences between them. It can also help characterize the risks and protective factors of subtypes of MDD and produce subpopulation-specific warning signals before relapse. The potential role of these mechanisms can then be tested in trans-diagnosed populations. (c) Methodological contributions. *First*, the proposed study will contribute to modifying the classical design for investigating mechanisms of change, into one that incorporates the premise that distinct subpopulations of patients may show different potential to benefit from any given mechanism, taking into account expected moderators in addition to mediators. *Second*, when testing the effects of mechanisms on outcome, studies have relied mostly on self-report measures, which depend heavily on the individual’s awareness, ability, and motivation to report on such processes. The present study uses interdisciplinary measures based on hormonal and acoustic measures, clinician interviews, and behavioral coding systems to complement the self-report measures. (d) Clinical contribution. The research on personalized mechanisms of change can help clinicians avoid unnecessary or irrelevant therapeutic elements, and focus on more efficient treatment delivery, adapted to given subpopulations of patients, according to their strengths and weaknesses. If a mechanism most likely to stimulate change for a subpopulation is identified, a corresponding treatment can be chosen that is most likely to activate that particular mechanism.

### Trial status

At the time of submission, recruitment status was pending: no participants are being recruited or enrolled yet. The anticipated date of enrolment of the first patient is November 2020. The recruitment phase of the trial is anticipated to be completed by end of 2026.

## Data Availability

Data sharing is not applicable to this article as no datasets were generated or analysed during the current study.

## Abbreviations

BAI: Beck anxiety inventory
BDI: Beck depression inventory
DERS: Difficulties in Emotion Regulation Scale
EFT: Emotion Focused Therapy
EACS: Emotion Approach Coping Scale
ERQ: Emotion Regulation Questionnaire
HRSD: Hamilton rating scale for depression
IIP: Inventory of interpersonal problems
MDD: Major depressive disorder
MINI: Mini International Neuropsychiatric Interview
MULTI: Multi-theoretical list of therapeutic interventions
OQ: Outcome questionnaire
PACS: Penn adherence-competence scale
PSQ: Post-session questionnaire
PANAS: Positive and Negative Affect Scale
PCEPS: Person-Centered and Experiential Psychotherapy Scale
Q-LES-S-: Quality of Life Enjoyment and Satisfaction Questionnaire
RCT: Randomized controlled trial
SE: Supportive-expressive
SIDP: Structured interview for DSM-IV personality
WAI: Working alliance inventory
